# Substance Use Disorder in a National Heart Failure Cohort: Risk for Hospital Readmission and Mortality

**DOI:** 10.3390/jcm15186974

**Published:** 2026-09-09

**Authors:** Samantha L. Yeung, Mengxi Wang, Mimi Lou, Brandon M. Wiley, Tien M. H. Ng

**Affiliations:** 1 USC Alfred E. Mann School of Pharmacy and Pharmaceutical Sciences, University of Southern California, Los Angeles, CA 90033, USA; samantly@usc.edu (S.L.Y.); mengxiwa@usc.edu (M.W.); mimilou@usc.edu (M.L.); 2 Los Angeles General Medical Center, Keck School of Medicine of USC, University of Southern California, Los Angeles, CA 90033, USA; bwiley@dhs.lacounty.gov

**Keywords:** heart failure, substance use, hospital readmission, mortality

## Abstract

**Background**: Heart failure (HF) and substance use disorder (SUD) are independently associated with morbidity and mortality. How SUD influences HF outcomes has not been described longitudinally in a national cohort. **Methods**: Retrospective study of adult patients with a primary diagnosis of HF enrolled in the Optum Clinformatics Data Mart (2010–2020), stratified by history of SUD (any time; prior or current use; substance type) compared to no SUD history. Logistic regression analyses were conducted in propensity score 1:1 matched (PSM) cohorts. Outcomes were 30-day, 180-day, and 1-year hospital readmission and mortality. **Results**: Study included 194,020 patients, 11,611 (6.0%) with documented SUD at any time. Prior to matching, patients with SUD were younger, more often male, and with fewer comorbidities compared to no history of SUD. In the PSM analysis, SUD was associated with greater 30-day [21.1% vs. 15.0%, *p* < 0.001], 180-day [54.7% vs. 41.9%, *p* < 0.001], and 1-year [67.9% vs. 55.4%, *p* < 0.001] hospital readmission. Thirty-day [1.6% vs. 1.0%, *p* < 0.001], 180-day [5.9% vs. 4.2%, *p* < 0.001] and 1-year [15.4% vs. 12.5%, *p* < 0.001] mortality was also higher in patients with SUD. In multivariable analyses, SUD remained independently associated with higher odds of poorer outcomes. Risk was retained when SUD was restricted to use prior to or at time of index hospitalization. Methamphetamine and other stimulant use were associated with an increased risk of hospital readmission across all time points, and an increased risk of mortality at one year. **Conclusions**: In patients with HF, SUD was independently associated with an increased risk of hospital readmissions and mortality.

## 1. Introduction

Heart failure (HF) continues to be a significant public health challenge that affects over 6 million individuals and contributes to over 800,000 hospital admissions annually in the United States (US) [1]. Despite advancements in evidence-based medical therapy and in the understanding of the pathophysiology behind this clinical syndrome, prognosis remains poor with an estimated five-year mortality rate of 50% [2]. Data from the Get with the Guidelines—Heart Failure registry from 2005 to 2014 found that patients admitted to the hospital for HF have been presenting with an increasing number of non-cardiovascular comorbidities and an associated worsening of outcomes over time [3]. Identifying and addressing these comorbidities will therefore be crucial to improving the clinical outcomes of these patients with HF.

Substance use disorders (SUDs) also contribute significantly to the public health burden and are associated with substantial morbidity and mortality. In 2019, the National Survey on Drug Use and Health demonstrated that 19.3 million individuals or 7.7% of the population in the US aged 18 years or older have an SUD [4]. Many drugs of misuse exert adverse effects on the cardiovascular system through mechanisms such as sympathetic nervous system stimulation, direct cardiomyocyte toxicity, and conduction system dysfunction, which can lead to the development of hypertension, coronary artery disease, arrhythmias, stroke, and HF [5,6]. Additionally, drugs of misuse have the potential to impair cognitive function and decision-making capabilities, which may impact cardiovascular outcomes through influences on adherence to medical therapy, socioeconomic determinants of health, and psychiatric disorders. In the US, there has been growing concern specifically over the methamphetamine epidemic and methamphetamine-associated cardiomyopathy (MACM) [7]. Most of the literature evaluating the impact of substance use on longitudinal clinical outcomes in HF has been limited to studies with short-term outcome data or from single-center sites [8,9,10]. Additionally, the literature describing how substance use influences HF prognosis on a national level is lacking. As a result, there is a critical need to further describe SUDs in HF, and to characterize the outcomes of these vulnerable patient populations.

In this study, we aimed to evaluate the influence of drugs of misuse on clinical outcomes (hospital readmission and mortality) in patients hospitalized with HF, expanding on the existing literature by examining outcomes over a greater longitudinal timeframe and utilizing data representing a national patient database. We hypothesized that substance use will be independently associated with greater risk of hospital readmission and mortality.

## 2. Methods

### 2.1. Study Design

This retrospective cohort study utilized pre-existing de-identified data on adult patients (≥18 years) enrolled in the Optum Clinformatics^®^ Data Mart, which covers 14 million privately insured and Medicare Advantage beneficiaries, from 1 January 2010 to 31 December 2020. The de-identified Clinformatics^®^ Data Mart (OptumInsight, Eden Prairie, MN, USA) is a large-scale dataset of patients with both medical and pharmacy coverage from a large commercial insurer in the US.

Adults with a primary diagnosis of HF with or without a diagnosis of active or prior SUD were included in this study. All diagnoses were identified by ICD-9 or -10 codes. The diagnosis of HF was not stratified by left ventricular ejection fraction (LVEF). Substance use was categorized by type and included: cannabis, opiates, cocaine, methamphetamine and other stimulants, and hallucinogens (phencyclidine and sedative/hypnotic/anxiolytics). History of alcohol use disorder was collected but not included in the SUD cohort as the effects of alcohol on cardiovascular risk can be variable [11,12]. However, to account for a possible influence of alcohol use disorder on the outcomes of interest, it was included as a covariate in the multivariable analysis (as described below). This study excluded patients who were pregnant or had less than 6 months of follow-up data. Participant informed consent was waived due to this study utilizing existing de-identified data. This study was approved as Exempt by the University of Southern California Institutional Review Board (#HS-22-00586).

Enrollment data on patient demographics (age, gender, race) were linked to longitudinal claims capturing admission and discharge dates, diagnosis of HF, type and history of substance use, mortality date, Elixhauser Comorbidity Index, and comorbid conditions. The Elixhauser Comorbidity Index is a well-validated scoring system to characterize the risk of in-hospital mortality using 31 comorbid conditions and a scoring system of −7 to +12, with higher scores indicating greater risk of mortality [13]. The primary diagnoses during hospital readmissions were also obtained.

Patients were stratified into cohorts of those with or without a diagnosis of SUD based on ICD-9/10 codes. Patient data were analyzed from index HF hospitalization to 12 months after discharge. The main outcomes of interest were 30-day, 180-day, and 1-year all-cause hospital readmission, HF-related hospital readmission (defined as a readmission with HF as the primary diagnosis), and mortality.

### 2.2. Statistical Analysis

The data were examined as a raw unadjusted cohort and as three propensity score matched (PSM) cohorts based on SUD history. In order to estimate the influence of substance use, the PSM cohorts matched patients with no history of SUD to patients (1) with SUD at any time, (2) in a subgroup with previous or current SUD, and (3) in a subgroup with methamphetamine and other stimulant use at any time. Methamphetamine use and HF is of particular interest amid a growing epidemic in the US. Additional characterization of MACM outcomes is needed, prompting the specific subgroup analysis of methamphetamine and other stimulant use [7,14].

As the cohort without substance use was substantially larger than the cohort with substance use, most of the baseline characteristics were significantly different. The first PSM analysis was conducted to consider the isolated effect of SUD considering the baseline differences in patient characteristics when stratified for history of SUD at any time. A one-to-one greedy matching method was applied to minimize the confounding due to measured covariates when estimating the influence of the presence of substance use at any time. This method involved matching pairs of substance users and non-substance users with the closest propensity score that lay within a fixed distance within a fixed caliper width of 0.3 to balance the difference in the baseline covariates [15]. Matching was performed without replacement to ensure that each comparison patient was matched only once. The propensity score for SUD at any time was estimated using a logistic regression, which examined the association of relevant covariates (age, gender, Elixhauser Comorbidity Index, and comorbid conditions) with the dichotomous dependent variable of substance use vs. no substance use.

The same PSM algorithm was applied for the other two subgroup cohorts: previous or current SUD, and methamphetamine and other stimulant use at any time. Baseline characteristics were reported as frequencies with percentages for categorical variables and means with standard deviations (SD) for continuous variables. Continuous and categorical variables were compared between the substance use and non-substance use cohorts using the Student’s t-test and the Chi-squared test, respectively. The Random Forest method (RF) as a supervised machine learning algorithm was employed in this study to perform both classification and regression prediction [16]. The clinical factors assessed as continuous or categorical variables (same as in the main analysis) with the potential to impact the outcomes were selected as the input to the RF ensembles. About 15–20 preselected factors from the RF ensembles, as one ensemble for each model outcome, were then evaluated in the logistic regressions in the order of their importance (i.e., ranking). The final multivariable logistic regression models only included significant predictors for the major endpoints (Supplementary Materials). A *p*-value of <0.05 was considered statistically significant and 95% confidence intervals were reported for all odds ratios. To complement the odds ratios obtained from multivariable logistic regression, relative risks (RRs) were estimated using modified Poisson regression with robust variance estimation, with the same covariates included in the corresponding multivariable logistic regression models. All statistical analyses were performed using SAS, version 9.4 (SAS Institute, Cary, NC, USA).

## 3. Results

### 3.1. Study Population

A total of 194,020 patients were identified with a history of HF, with 6.0% (*n* = 11,611) having an ICD-9/10 code for a history of SUD at any time (Table 1). The mean age of the population was 75 ± 11 years, with 51.0% being female sex, and 60.3% of Caucasian race/ethnicity. Patients with a history of SUD were younger (68 ± 12 years) and more often of male sex (51.5%) compared to those with no history of SUD. There was a greater proportion of patients of African American and Hispanic ethnicity in those with SUD, although Caucasian remained the most predominant. The SUD cohort had a lower comorbidity burden compared to those with no history of SUD as defined by the Elixhauser Comorbidity Index. With respect to cardiovascular disease, patients with SUD had a higher percentage of diabetes whereas patients without SUD were more likely to have hypertension, stroke, or dysrhythmia. There was no difference in ischemic heart disease and hyperlipidemia. Patients with SUD also had a greater proportion of alcohol and tobacco use disorder. Index hospitalization length of stay was similar between the substance use and no substance use cohorts; however, a higher percentage of those with a history of SUD were admitted to an intensive care unit (ICU).

The unadjusted data showed substantial differences in outcomes between patients with a history of any SUD at any time versus those with no history of SUD (Supplementary Materials). Those with a history of SUD had a higher incidence of all-cause hospital readmission at 30 days (21.1% vs. 14.7%), 180 days (54.7% vs. 41.9%), and at one year (67.9% vs. 56.4%). HF-related hospital readmission was also higher at 30 days (6.6% vs. 4.9%), 180 days (21.4% vs. 16.2%), and at one year (28.8% vs. 23.1%) in patients with a history of SUD. All-cause mortality assessed at 30 days (1.6% vs. 1.3%) and 180 days (5.9% vs. 5.4%) was also higher in patients with a history of SUD compared to no history of SUD. The higher mortality rate was not evident at one year (15.4% vs. 16.1%). When stratified for timing of documented SUD by limiting to prior to or at the time of index hospitalization, the incidence of all-cause and HF-related hospital readmissions remained higher compared to no history of SUD in both strata.

### 3.2. Propensity Score Matched Analysis for Substance Use at Any Time and Outcomes

To further isolate and explore the relationship between SUDs and longitudinal outcomes in patients with HF, an analysis was conducted with a comparator group of patients with HF and no history of SUD after PSM. After matching, two cohorts of 11,611 patients were balanced in terms of mean age (68 ± 12 years vs. 68 ± 13 years), female sex (48.5% vs. 49.7%), and Elixhauser comorbidity index score (1.0 ± 3.6 vs. 0.8 ± 3.2) (Table 2).

In the simple logistic regression analysis, history of SUD at any time was associated with 1.5-fold higher odds of all-cause hospital readmission at 30 days and 1.7-fold higher odds at 180 days and one year. HF-related hospital readmission was also approximately 1.5-fold higher at each time point (Table 3). Readmissions related to SUD were rare (<1%) (Supplementary Materials). All-cause mortality was higher at 30 days (1.6-fold), 180 days (1.4-fold), and remained significant at one year (1.3-fold) in patients with a history of SUD compared to those with no history of SUD. When a multivariable analysis was applied, SUD remained significantly associated with a higher odds of both hospital readmission and mortality (Table 4 and Supplementary Materials). Renal disease was also independently associated with higher odds of both hospital readmission and mortality.

To account for patients who had less than one full year of follow-up and no events being included in the cohort that may bias towards fewer events, a sensitivity analysis was conducted limiting the cohort to patients with a full year of follow-up. In this analysis, the associations to increased risk were retained and similar in magnitude (Supplementary Materials).

When SUD was stratified for patients with only one drug of misuse compared to more than one drug of misuse (i.e., polysubstance use), polysubstance use was associated with higher odds of hospital readmissions at 30 days, 180 days, and one year. Mortality did not differ significantly at 30 or 180 days; however, polysubstance use was associated with significantly lower mortality at one year compared with one drug of misuse (Supplementary Materials).

### 3.3. Propensity Score Matched Analysis for Subgroup of Prior or Current Substance Use and Outcomes

To factor for temporal relation where SUD was documented at baseline, a subgroup analysis was performed to restrict the cohort to patients with SUD documented prior to or at the time of index hospitalization. After matching, two cohorts of 7069 patients were balanced (Table 5). When history of SUD was examined by timing, history limited to prior to or at the time of index hospitalization remained associated with a higher odds of 30-day, 180-day, and 1-year all-cause (1.3 to 1.4-fold higher odds) and HF-related (1.1 to 1.3-fold higher odds) hospital readmissions compared to those with no history of SUD (Table 6). The risk of all-cause mortality was also higher in the cohort with a history of SUD prior to or at index hospitalization (1.3 to 2.0-fold higher odds). In the sensitivity analysis limited to patients with one full year of follow-up, the associations to increased risk were retained for all-cause readmissions and mortality, but not for HF-related readmissions (Supplementary Materials).

### 3.4. Propensity Score Matched Analysis for Subgroup of Methamphetamine and Other Stimulant Use and Outcomes

Using a cohort that was PSM based on a history of methamphetamine and other stimulant use alone, there was a significant and consistent (1.5 to 2.2-fold) increased odds of 30-day, 180-day, and 1-year all-cause and HF-related hospital readmission (Table 7). History of methamphetamine and other stimulant use was also associated with higher odds of all-cause mortality at one year, but not at 30 and 180 days. The characteristics of the PSM cohorts based on methamphetamine and other stimulant use are provided in the Supplementary Materials.

## 4. Discussion

Substance use disorders are recognized as significant contributors of morbidity and short-term mortality in patients with HF. Beyond the direct adverse cardiovascular effects of drugs of misuse, substance use can affect many aspects of an individual’s life that may influence patient outcomes. It may impair judgement regarding health-related behaviors and compromise cognitive functioning essential for medication adherence and attendance to follow-up appointments [17]. The stigma of SUD may also influence practitioners and healthcare delivery [18]. Most of the literature in this area, however, has been limited to studies with short-term outcome data or from single-center sites [8,9,10]. Our study, utilizing data from a large national database, demonstrated that 6.0% of patients hospitalized with a primary diagnosis of HF had a history of SUD. It also reveals that SUDs are associated with a substantial increased risk of hospitalizations and mortality that is evident well beyond the hospital stay. Substance use disorder was consistently associated with increased hospital readmissions across 30-day, 180-day, and 1-year timeframes, and with mortality. This increased risk was evident even after considering (PSM) and adjusting (covariance) for relevant differences in patient and clinical characteristics of the two populations. Using a large national patient database, this study expands the current literature by examining the influence of SUD across multiple longitudinal time points out to one year.

This study adds to the current understanding of SUDs in patients with HF. A single-center study of 11,268 adults at an academic medical center in California found that 15.2% of patients with HF with an average age of 55 years were diagnosed with substance abuse [8]. Substance abuse was independently associated with emergency department visits and hospitalizations for HF, but dissimilar to our study, was not associated with all-cause mortality. A retrospective cohort study of 57,985 young adults aged 18–39 years utilizing medical records from the 2019 National Inpatient Sample database identified substance use in 20.9% of patients admitted with cardiac events [9]. Despite a lower prevalence of traditional cardiovascular risk factors compared to patients without SUDs, SUD was significantly associated with HF (OR 2.6 (95% CI 2.4–3.0), *p* < 0.001) and in-hospital mortality (OR = 1.5 (95% CI 1.2–2.0), *p* < 0.001). However, the analysis was only limited to in-hospital outcomes. Our analysis extends these findings to include 30-day, 180-day, and 1-year outcomes. Furthermore, our study includes a more contemporary, larger and older patient population. The reported prevalence of substance use in our study was lower than reported previously, but this may reflect the fact that ICD coding was used to identify a history of SUD as opposed to a social history of substance use.

Although the presence of an SUD was associated with both higher rates of hospital readmissions and mortality in the PSM cohorts, the association with mortality was less consistent in the unadjusted analysis. In the unadjusted analysis, 30-day and 180-day mortality were significantly higher in patients with a history of SUD but this was not evident at one year. The difference between the unadjusted and PSM cohorts for 1-year mortality is likely due to the differing demographics between those with an SUD history and those without in the unadjusted overall study population. In the overall study population, patients with a history of SUD were younger and had a lower comorbidity burden compared to those with no history of SUD. There were also differences in the incidence of certain cardiovascular risk factors and diseases between patients with or without a history of SUD. Therefore, there are likely additional factors contributing to the mortality risk. The complexity of factors influencing outcomes of these patients is also highlighted in the finding that SUD limited to the subgroup of previous or current use, removing patients who purportedly ceased use, was associated with an elevated risk of all-cause hospitalizations but risk of HF-related hospitalizations was numerically lower, suggesting patients may be seeking medical care for other non-cardiovascular-related conditions.

Of particular interest is the impact of stimulants such as methamphetamine on clinical outcomes in patients with HF, particularly the outcomes of patients with MACM [7,14,19]. Methamphetamine is believed to cause both direct and indirect myocardial damage through multiple pathological mechanisms, including oxidative stress, apoptosis, mitochondrial dysfunction, inflammation, and fibrosis [7]. It is recognized that MACM is characterized by more severe disease and poorer short-term outcomes, though there is a potential for cardiac function recovery with sustained abstinence from methamphetamine. However, the existing literature on MACM has been limited to small single-center studies. While a retrospective case series conducted in New Zealand reported the longitudinal outcomes of 30 patients with HF and a documented history of either current or past amphetamine use, the study did not include a comparator group [20]. Another retrospective case series from New Zealand compared outcomes for patients with HF and a documented history of either current or past methamphetamine use to a matched cohort not related to ischemic heart disease, methamphetamine, alcohol, or other recreational drug use [21]. Although longitudinal outcomes were reported, the study was limited to only 62 patients. A 2019 study reviewing the medical records of 106 HF patients who use methamphetamine and 96 HF patients without evidence of methamphetamine use from a Veterans Affairs Medical Center in California found that at 180 days, emergency department or HF readmission was greater in the methamphetamine cohort but was not statistically significant (log-rank *p* = 0.34) [22]. Similar to our study, the HF patients who used methamphetamine were younger compared to the HF patients who did not use methamphetamine (61 ± 7 years vs. 71 ± 12 years). A retrospective cohort study reviewing the electronic medical records of 896 patients with both HF and methamphetamine abuse, and 20,576 patients with HF with no evidence of methamphetamine abuse from a single academic medical center in California, found that patients with methamphetamine abuse had a higher risk of HF readmission (log rank *p* < 0.001) and a lower risk of total mortality (log rank *p* < 0.001) [23]. Once again, patients with both HF and methamphetamine abuse were younger than the patients with HF with no evidence of methamphetamine abuse (50 ± 10 years vs. 67 ± 16 years). The current study found that methamphetamine users had a significant and consistent increase in 30-day, 180-day, and 1-year all-cause and HF-related hospital readmission. The results of our study are similar to a prior study from a single health system conducted from 2001 and 2019 showing that individuals with methamphetamine use who have a median age of 52 years are at an increased risk of hospital readmission at 30 days, 90 days, and one year [24]. Our study, however, encompasses an older patient population and reinforces these findings in a national cohort. A recent case-control study of two academic medical emergency departments found patients with MACM experienced more emergency department visits and hospitalizations and had longer lengths of stay [25]. Unfortunately, the retrospective nature of the data does not allow for clear determination of whether these patients had MACM.

The study findings have important implications. Whether the poorer outcomes reflect pathophysiologic mechanisms contributing to further or accelerated myocardial damage and/or socioeconomic barriers remains to be studied further. In lieu of this data, along with HF-specific therapy optimization, there is a need for routine screening for SUDs in patients with HF and supporting programs that promote the cessation of substance use [19] and integrated care models (NCT07211724).

### Limitations

There are limitations to the current study that warrant mention. First, the retrospective, observational study design does not allow for true determination of cause and effect, although mechanisms affecting the cardiovascular system have been previously described for the various drugs of misuse [7,26,27,28]. Second, our study utilized claims data to investigate the research question. Inherent limitations of utilizing claims data have been well described [29,30,31]. Claims data are dependent on ICD coding. Studies have shown that in the clinical setting, some diagnoses may be missed as different professions may have different coding patterns and claim submission practices change over time. Additionally, undercoding, differential detection across clinical settings, and changes associated with the transition from ICD-9 to ICD-10 coding during the study period may have contributed to the misclassification of exposure status. As such, there may be an underreporting of data. However, the Optum Clinformatics^®^ Data Mart provides an opportunity to obtain a large sample representative of patients with HF on a national level. Third, our study is unable to classify HF based on LVEF and therefore does not stratify HF with reduced EF from HF with preserved EF. Fourth, the Optum Clinformatics^®^ Data Mart database lacks patient-level clinical data. Therefore, the current analyses were not able to assess or adjust for specific HF characteristics and laboratory parameters that are linked to HF prognosis. While this is a limitation, this database provides a national representative sample and the statistical methods were rigorous in controlling for patient characteristics and overall disease burden validated to predict mortality, readmissions, and healthcare resource utilization [13]. Lastly, the outcomes reflect an insured patient population in the United States and may not apply to patients in other parts of the world.

## 5. Conclusions

Concomitant history of SUD is independently associated with an increased risk of all-cause and HF-related hospital readmissions and mortality in patients with HF. Early recognition and timely treatment of SUDs may help improve outcomes and reduce healthcare utilization among patients with HF. Future research should focus on identifying and developing integrated care models with targeted interventions aimed at addressing SUDs in patients with HF.

## Figures and Tables

**Table 1:** Baseline characteristics of study population by history of SUD.

Baseline Characteristics	Total Study Population (*n* = 194,020)	Substance Use at Any Time (*n* = 11,611)	No History of Substance Use (*n* = 182,409)	*p*-Value *^a^*
Age, mean ± SD	74.8 ± 11.1	68.3 ± 11.9	75.2 ± 11.0	<0.001
Female Sex, *n* (%)	98,980 (51.0%)	5634 (48.5%)	93,346 (51.2%)	<0.001
Race/Ethnicity, *n* (%)				<0.001
Caucasian	116,986 (60.3%)	6291 (54.2%)	110,695 (60.7%)	
African American	31,783 (16.4%)	2178 (18.8%)	29,605 (16.2%)	
Hispanic	17,275 (8.9%)	1290 (11.1%)	15,985 (8.8%)	
Asian	3678 (1.9%)	147 (1.3%)	3531 (1.9%)	
Other	24,298 (12.5%)	1705 (14.7%)	22,593 (12.4%)	
Elixhauser Comorbidity Index, mean ± SD	3.0 ± 5.6	1.0 ± 3.6	3.2 ± 5.7	<0.001
Comorbid Conditions, *n* (%)				
Diabetes Type 1 or 2	89,415 (46.1%)	5658 (48.7%)	83,757 (45.9%)	<0.001
Hypertension	80,837 (41.7%)	4328 (37.3%)	76,509 (41.9%)	<0.001
Hyperlipidemia	99,837 (51.5%)	5986 (51.6%)	93,851 (51.5%)	0.83
Ischemic Heart Disease	101,437 (52.3%)	6016 (51.8%)	95,421 (52.3%)	0.30
Cardiac Dysrhythmia	85,569 (44.1%)	4388 (37.8%)	81,181 (44.5%)	<0.001
Stroke	3195 (1.6%)	140 (1.2%)	3055 (1.7%)	<0.001
COPD	48,694 (25.1%)	4651 (40.1%)	44,043 (24.1%)	<0.001
Asthma	12,420 (6.4%)	872 (7.5%)	11,548 (6.3%)	<0.001
Renal Disease	99,944 (51.5%)	5994 (51.6%)	93,950 (51.5%)	0.80
Liver Disease	3992 (2.1%)	468 (4.0%)	3524 (1.9%)	<0.001
Cancer	8681 (4.5%)	486 (4.2%)	8195 (4.5%)	0.12
Anemia	6460 (3.3%)	482 (4.2%)	5978 (3.3%)	<0.001
Obesity	36,907 (19.0%)	3020 (26.0%)	33,887 (18.6%)	<0.001
Sleep Apnea	28,323 (14.6%)	2438 (21.0%)	25,885 (14.2%)	<0.001
Tobacco Use Disorder	16,747 (8.6%)	2748 (23.7%)	13,999 (7.7%)	<0.001
Alcohol Use	3870 (2.0%)	630 (5.4%)	3240 (1.8%)	<0.001
Length of Stay, mean ± SD	6.47 ± 14.09	6.35 ± 8.53	6.47 ± 14.38	0.364
ICU, *n* (%)	66,315 (34.3%)	4398 (38.0%)	61,917 (34.1%)	<0.001

COPD: chronic obstructive pulmonary disease, ICU: intensive care unit, SD: standard deviation. *^a^ p*-value < 0.05 indicates statistical significance.

**Table 2:** Baseline characteristics of study population before and after PSM based on history of SUD at any time.

	Unmatched			Matched		
Baseline Characteristics	Substance Use at Any Time (*n* = 11,611)	No History of Substance Use (*n* = 182,409)	Standardized Differences	Substance Use at Any Time (*n* = 11,611)	No History of Substance Use (*n* = 11,611)	Standardized Differences
Age, mean ± SD	68.3 ± 11.9	75.2 ± 11.0	0.603	68.3 ± 11.9	68.4 ± 12.7	0.006
Female Sex, *n* (%)	5634 (48.5%)	93,346 (51.2%)	0.053	5634 (48.5%)	5772 (49.7%)	0.024
Elixhauser Comorbidity Index, mean ± SD	1.0 ± 3.6	3.2 ± 5.7	0.455	1.0 ± 3.6	0.8 ± 3.2	0.059
Comorbid Conditions, *n* (%)						
Diabetes Type 1 or 2	5658 (48.7%)	83,757 (45.9%)	0.056	5658 (48.7%)	5675 (48.9%)	0.003
Hypertension	4328 (37.3%)	76,509 (41.9%)	0.096	4328 (37.3%)	4265 (36.7%)	0.011
Hyperlipidemia	5986 (51.6%)	93,851 (51.5%)	0.002	5986 (51.6%)	5964 (51.4%)	0.004
Ischemic Heart Disease	6016 (51.8%)	95,421 (52.3%)	0.01	6016 (51.8%)	5979 (51.5%)	0.006
Cardiac Dysrhythmia	4388 (37.8%)	81,181 (44.5%)	0.137	4388 (37.8%)	4330 (37.3%)	0.01
Stroke	140 (1.2%)	3055 (1.7%)	0.039	140 (1.2%)	93 (0.8%)	0.041
COPD	4651 (40.1%)	44,043 (24.1%)	0.346	4651 (40.1%)	4650 (40.0%)	0
Asthma	872 (7.5%)	11,548 (6.3%)	0.046	872 (7.5%)	750 (6.5%)	0.041
Renal Disease	5994 (51.6%)	93,950 (51.5%)	0.002	5994 (51.6%)	5960 (51.3%)	0.006
Liver Disease	468 (4.0%)	3524 (1.9%)	0.124	468 (4.0%)	349 (3.0%)	0.056
Cancer	486 (4.2%)	8195 (4.5%)	0.015	486 (4.2%)	380 (3.3%)	0.048
Anemia	482 (4.2%)	5978 (3.3%)	0.046	482 (4.2%)	403 (3.5%)	0.036
Obesity	3020 (26.0%)	33,887 (18.6%)	0.179	3020 (26.0%)	3165 (27.3%)	0.028
Sleep Apnea	2438 (21.0%)	25,885 (14.2%)	0.179	2438 (21.0%)	2494 (21.5%)	0.012
Tobacco Use Disorder	2748 (23.7%)	13,999 (7.7%)	0.451	2748 (23.7%)	2612 (22.5%)	0.028
Alcohol Use	630 (5.4%)	3240 (1.8%)	0.197	630 (5.4%)	549 (4.7%)	0.032

COPD: chronic obstructive pulmonary disease, SD: standard deviation.

**Table 3:** All-cause and HF-related hospital readmission and death of cohorts after PSM based on history of SUD at any time.

	Total Population (*n* = 23,222)	Substance Use at Any Time (*n* = 11,611)	No History of Substance Use (*n* = 11,611)	Odds Ratio	Relative Risk	95% OR CI	OR *p*-Value *^a^*
30-day Hospital Readmission, *n* (%)							
All-cause	4190 (18%)	2454 (21.1%)	1736 (15%)	1.5	1.4	1.4–1.6	<0.001
HF-related	1293 (5.6%)	772 (6.6%)	521 (4.5%)	1.5	1.5	1.4–1.7	<0.001
180-day Hospital Readmission, *n* (%)							
All-cause	11,219 (48.3%)	6350 (54.7%)	4869 (41.9%)	1.7	1.3	1.6–1.8	<0.001
HF-related	4339 (18.7%)	2485 (21.4%)	1854 (16%)	1.4	1.3	1.3–1.5	<0.001
1-year Hospital Readmission, *n* (%)							
All-cause	14,314 (61.6%)	7881 (67.9%)	6433 (55.4%)	1.7	1.2	1.6–1.8	<0.001
HF-related	6006 (25.9%)	3345 (28.8%)	2661 (22.9%)	1.4	1.3	1.3–1.4	<0.001
Mortality, *n* (%)							
30-day Mortality	308 (1.3%)	191 (1.6%)	117 (1.0%)	1.6	1.6	1.3–2.1	<0.001
180-day Mortality	1174 (5.1%)	681 (5.9%)	493 (4.2%)	1.4	1.4	1.2–1.6	<0.001
1-year Mortality	3249 (14%)	1793 (15.4%)	1456 (12.5%)	1.3	1.2	1.2–1.4	<0.001

CI: confidence interval, HF: heart failure. *^a^ p* value < 0.05 indicates statistical significance.

**Table 4:** Summary of SUD at any time as a major factor in multivariable logistic regressions associated with readmissions and mortality at 30 days and 1 year *.

	Odds Ratio	95% CI	*p*-Value *^a^*
30-day All-cause Hospital Readmission	1.5	1.4–1.6	<0.001
30-day HF-related Hospital Readmission	1.5	1.4–1.7	<0.001
1-year All-cause Hospital Readmission	1.7	1.6–1.8	<0.001
1-year HF-related Hospital Readmission	1.4	1.3–1.5	<0.001
30-day All-cause Mortality	1.7	1.3–2.1	<0.001
1-year All-cause Mortality	1.3	1.2–1.4	<0.001

* Detailed multivariable models with other covariates are in the Supplementary Materials. CI: confidence interval, HF: heart failure. *^a^ p* value < 0.05 indicates statistical significance.

**Table 5:** Baseline characteristics of study population before and after PSM based on subgroup with previous history or current SUD.

	Unmatched			Matched		
Baseline Characteristics	Substance Use Prior/At HF Admission (*n* = 7069)	No Substance Use (*n* = 182,409)	Standardized Differences	Substance Use Prior/At HF Admission (*n* = 7069)	No Substance Use (*n* = 7069)	Standardized Differences
Age, mean ± SD	69.3 (±11.8)	75.0 (±11.1)	0.501	69.3 (±11.8)	69.5 (±12.4)	0.017
Female Sex, *n* (%)	3543 (50.1%)	95,437 (51.0%)	0.019	3543 (50.1%)	3562 (50.4%)	0.005
Elixhauser Comorbidity Index, mean ± SD	1.0 (±3.6)	3.1 (±5.7)	0.449	1.0 (±3.6)	0.8 (±3.3)	0.048
Comorbid Conditions, *n* (%)						
Diabetes Type 1 or 2	3457 (48.9%)	85,958 (46.0%)	0.059	3457 (48.9%)	3536 (50.0%)	0.022
Hypertension	2588 (36.6%)	78,249 (41.9%)	0.108	2588 (36.6%)	2541 (35.9%)	0.014
Hyperlipidemia	3633 (51.4%)	96,204 (51.5%)	0.001	3633 (51.4%)	3680 (52.1%)	0.013
Ischemic Heart Disease	3650 (51.6%)	97,787 (52.3%)	0.013	3650 (51.6%)	3697 (52.3%)	0.013
Cardiac Dysrhythmia	2760 (39.0%)	82,809 (44.3%)	0.107	2760 (39.0%)	2730 (38.6%)	0.009
Stroke	95 (1.3%)	3100 (1.7%)	0.026	95 (1.3%)	72 (1.0%)	0.03
COPD	2887 (40.8%)	45,807 (24.5%)	0.354	2887 (40.8%)	2907 (41.1%)	0.006
Asthma	534 (7.6%)	11,886 (6.4%)	0.047	534 (7.6%)	442 (6.3%)	0.051
Renal Disease	3669 (51.9%)	96,275 (51.5%)	0.008	3669 (51.9%)	3678 (52.0%)	0.003
Liver Disease	261 (3.7%)	3731 (2.0%)	0.102	261 (3.7%)	215 (3.0%)	0.036
Cancer	309 (4.4%)	8372 (4.5%)	0.005	309 (4.4%)	228 (3.2%)	0.06
Anemia	302 (4.3%)	6158 (3.3%)	0.051	302 (4.3%)	280 (4.0%)	0.016
Obesity	1863 (26.4%)	35,044 (18.7%)	0.183	1863 (26.4%)	1888 (26.7%)	0.008
Sleep Apnea	1503 (21.3%)	26,820 (14.3%)	0.182	1503 (21.3%)	1499 (21.2%)	0.001
Tobacco Use Disorder	1580 (22.4%)	15,167 (8.1%)	0.404	1580 (22.4%)	1544 (21.8%)	0.012
Alcohol Use	385 (5.4%)	3485 (1.9%)	0.192	385 (5.4%)	355 (5.0%)	0.019

COPD: chronic obstructive pulmonary disease, SD: standard deviation.

**Table 6:** All-cause and HF-related hospital readmission and death of cohorts after PSM based on subgroup with previous history or current SUD.

	Total Population (*n* = 14,138)	Substance Use Prior/at HF Admission (*n* = 7069)	No History of Substance Use (*n* = 7069)	Odds Ratio	Relative Risk	95% OR CI	OR *p*-Value *^a^*
30-day Hospital Readmission, *n* (%)							
All-cause	2243 (16.2%)	1285 (18.2%)	958 (14.2%)	1.3	1.1	1.2–1.5	<0.001
HF-related	689 (5.0%)	400 (5.7%)	289 (4.3%)	1.3	1.2	1.1–1.6	<0.001
180-day Hospital Readmission, *n* (%)							
All-cause	6240 (45.2%)	3435 (48.6%)	2805 (41.6%)	1.3	1.1	1.2–1.4	<0.001
HF-related	2399 (17.4%)	1320 (18.7%)	1079 (16.0%)	1.2	1.1	1.1–1.3	<0.001
1-year Hospital Readmission, *n* (%)							
All-cause	8253 (59.7%)	4475 (63.3%)	3778 (56.0%)	1.4	1.1	1.3–1.5	<0.001
HF-related	3418 (24.7%)	1830 (25.9%)	1588 (23.5%)	1.1	1.1	1.1–1.2	0.001
Mortality, *n* (%)							
30-day Mortality	254 (1.8%)	172 (2.4%)	82 (1.2%)	2.0	2.1	1.6–2.6	<0.001
180-day Mortality	802 (5.8%)	489 (6.9%)	313 (4.6%)	1.5	1.5	1.3–1.8	<0.001
1-year Mortality	2046 (14.8%)	1144 (16.2%)	902 (13.4%)	1.3	1.2	1.1–1.4	<0.001

CI: confidence interval, HF: heart failure. *^a^ p* value < 0.05 indicates statistical significance.

**Table 7:** All-cause and HF-related hospital readmissions and death of cohort after PSM based on history of methamphetamine and other stimulant use at any time.

	Total Population (*n* = 1936)	Methamphetamine and Other Stimulant Use at Any Time (*n* = 968)	No History of Substance Use (*n* = 968)	Odds Ratio	Relative Risk	95% OR CI	OR *p*-Value *^a^*
30-day Hospital Readmission, *n* (%)							
All-cause	383 (19.8%)	251 (25.9%)	132 (13.6%)	2.2	1.9	1.8–2.8	<0.001
HF-related	125 (6.5%)	78 (8.1%)	47 (4.9%)	1.7	1.7	1.2–2.5	0.01
180-day Hospital Readmission, *n* (%)							
All-cause	994 (51.3%)	587 (60.6%)	407 (42.0%)	2.1	1.4	1.8–2.5	<0.001
HF-related	424 (21.9%)	248 (25.6%)	176 (18.2%)	1.5	1.4	1.2–1.9	<0.001
1-year Hospital Readmission, *n* (%)							
All-cause	1208 (62.4%)	670 (69.2%)	538 (55.6%)	1.8	1.2	1.5–2.2	<0.001
HF-related	568 (29.3%)	323 (33.4%)	245 (25.3%)	1.5	1.3	1.2–1.8	<0.001
Mortality, *n* (%)							
30-day Mortality	22 (1.1%)	15 (1.5%)	7 (0.7%)	2.2	2.1	0.9–5.3	0.10
180-day Mortality	78 (4.0%)	46 (4.8%)	32 (3.3%)	1.5	1.4	0.9–2.3	0.12
1-year Mortality	231 (11.9%)	133 (13.7%)	98 (10.1%)	1.4	1.4	1.1–1.9	0.01

CI: confidence interval, HF: heart failure. *^a^ p* value < 0.05 indicates statistical significance.

## Data Availability

The data were available under an institutional data use agreement with OptumInsight, Eden Prairie, MN, USA.

## Supplementary Materials

The following supporting information can be downloaded at: https://www.mdpi.com/article/10.3390/jcm15186974/s1, Table S1: ICD-9/10 codes for substance use disorders; Table S2: Unadjusted all-cause and HF-related readmissions and death based on history of SUD at any time; Table S3: Multivariable models for 30-day all-cause and HF-related hospital readmission after PSM based on history of SUD at any time; Table S4: Multivariable models for 1-year all-cause and HF-related hospital readmission after PSM based on history of SUD at any time; Table S5: Multivariable model for 30-day and 1-year all-cause mortality after PSM based on history of SUD at any time; Table S6: All-cause and HF-related hospital readmissions, and death of cohort after PSM based on history of SUD at any time and patients with one full year follow-up; Table S7: All-cause and HF-related hospital readmissions, and death of cohort after PSM based on subgroup with previous history or current SUD and one full year of follow-up; Table S8: All-cause and HF-related hospital readmissions, and death of cohort after PSM based on history of polysubstance or mono-substance use at any time; Table S9; Baseline characteristics after PSM based on subgroup of methamphetamine and other stimulant use; Table S10: Readmissions with heart failure or substance use primary diagnosis in the substance use at any time matched cohort; Table S11: Readmissions with heart failure or substance use primary diagnosis in the prior/current substance use matched cohort; Table S12: Readmissions with heart failure or substance use primary diagnosis in the methamphetamine and other stimulant use at any time matched cohort; Figure S1: Love plot of covariate balance before and after propensity score matching for history of substance use at any time; Figure S2: Love plot of covariate balance before and after propensity score matching for prior or current substance use; Figure S3: Love plot of covariate balance before and after propensity score matching for history of methamphetamine use at any time.

## Abbreviations

The following abbreviations are used in this manuscript:

HF	Heart failure
ICU	Intensive care unit
LVEF	Left ventricular ejection fraction
MACM	Methamphetamine-associated cardiomyopathy
PSM	Propensity score matched
SD	Standard deviation
SUD	Substance use disorder
US	United States
