# Supplementary material for: Substance Use Disorder in a National Heart Failure Cohort: Risk for Hospital Readmission and Mortality
Source: J Clin Med. 2026 Sep 9;15(18):6974. doi: 10.3390/jcm15186974 (PMC13607335; doi:10.3390/jcm15186974)

Supplemental Appendix

Substance Use Disorder in a National Heart Failure Cohort: Risk for Hospital Readmission and Mortality

Table S1. ICD-9/10 codes for substance use disorders.

Category	ICD-9	ICD-10
Cannabis Use Disorder	304.3x, 305.2x	F12.x
Cocaine Use Disorder	304.2x, 305.6x, 970.81	F14.x
Hallucinogen Use Disorder	304.5x, 305.3x	F16.x
Methamphetamine Use Disorder	304.4x, 305.7x, 969.72	F15.x
Opioid Use Disorder	304.0x, 304.7x, 305.5x	F11.x
Sedative/hypnotic/anxiolytic Use Disorder	304.1x, 305.4x	F13.x

Table S2. Unadjusted all-cause and HF-related readmissions and death based on history of SUD at any time.

	**Total Population (n=194,020)**	**Substance Use at Any time (n=11,611)**	**No History of Substance Use (n=182,409)**	**P-value***	**Odds Ratio**	**95% CI**
**30-day Hospital Readmission,** n (%)						
**All-cause**	29355 (15.1%)	2454 (21.1%)	26901 (14.7%)	<0.001	1.5	1.5–1.6
**HF-related**	9620 (5.0%)	772 (6.6%)	8848 (4.9%)	<0.001	1.4	1.3–1.5
**180-day Hospital Readmission,** n (%)						
**All-cause**	82839 (42.7%)	6350 (54.7%)	76489 (41.9%)	<0.001	1.7	1.6–1.7
**HF-related**	32007 (16.5%)	2485 (21.4%)	29522 (16.2%)	<0.001	1.4	1.3–1.5
**1-year Hospital Readmission,** n (%)						
**All-cause**	110802 (57.1%)	7881 (67.9%)	102921 (56.4%)	<0.001	1.6	1.6–1.7
**HF-related**	45511 (23.5%)	3345 (28.8%)	42166 (23.1%)	<0.001	1.3	1.3–1.4
**Mortality,** n (%)						
**30-day Mortality**	2573 (1.3%)	191 (1.6%)	2382 (1.3%)	0.002	1.3	1.1–1.5
**180-day Mortality**	10477 (5.4%)	681 (5.9%)	9796 (5.4%)	0.022	1.1	1.0–1.2
**1-year Mortality**	31116 (16.0%)	1793 (15.4%)	29323 (16.1%)	0.076	1.0	0.9–1.0

* P value <0.05 indicates statistical significance.

Table S3. Multivariable models for 30-day All-cause and HF-related hospital readmission after PSM based on history of SUD at any time.

	30-day All-cause Hospital Readmission				30-day HF-related Hospital Readmission			
**Variable, n (%)**	**Odds Ratio**	**Relative Risk**	**95% CI**	**P value***	**Odds Ratio**	**Relative Risk**	**95% CI**	**P value***
All Time Substance Use	1.5	1.4	1.4-1.6	<0.0001	1.5	1.5	1.4-1.7	<0.0001
Age	1.0	1.0	1.0-1.0	<0.0001	1.0	1.0	1.0-1.0	<0.0001
Female	--	—	--	--	0.9	0.9	0.8-1.0	0.03
Alcohol Use	0.8	0.8	0.7-0.9	0.002	--	—	--	--
Cardiac Dysrhythmia	1.2	1.1	1.1-1.2	<0.001	1.2	1.2	1.1-1.4	<0.001
COPD	1.3	1.2	1.2-1.3	<0.0001	1.3	1.3	1.2-1.5	<0.0001
Diabetes Type 1 or 2	1.1	1.1	1.0-1.2	0.01	1.2	1.2	1.0-1.3	0.01
Hypertension	0.9	0.9	0.8-1.0	0.005	0.8	0.8	0.7-0.9	<0.001
Liver Disease	1.4	1.3	1.1-1.6	<0.001	--	—	--	--
Renal Disease	1.3	1.3	1.3-1.4	<0.0001	1.6	1.5	1.4-1.8	<0.0001
Sleep Apnea	0.8	0.9	0.8-0.9	<0.0001	0.8	0.8	0.7-1.0	0.01

* P value <0.05 indicates statistical significance.

Table S4. Multivariable models for 1-year All-cause and HF-related hospital readmission after PSM based on history of SUD at any time.

	1-year All-cause Hospital Readmission				1-year HF-related Hospital Readmission			
**Variable, n (%)**	**Odds Ratio**	**Relative Risk**	**OR 95% CI**	**OR P value***	**Odds Ratio**	**Relative Risk**	**95% CI**	**OR P value***
All Time Substance Use	1.7	1.2	1.6–1.8	<0.0001	1.4	1.3	1.3–1.5	<0.0001
Female	1.1	1.1	1.0–1.1	0.04	--	—	--	--
Alcohol Use	0.8	0.9	0.7–0.9	0.001	0.9	0.9	0.7–1.0	0.03
Anemia	1.4	1.1	1.2–1.6	<0.0001	--	—	--	--
Cardiac Dysrhythmia	1.1	1	1.1–1.2	<0.0001	1.1	1.1	1.1–1.2	<0.001
COPD	1.4	1.1	1.3–1.5	<0.0001	1.4	1.3	1.3–1.5	<0.0001
Diabetes Type 1 or 2	1.4	1.1	1.3–1.5	<0.0001	1.3	1.2	1.2–1.4	<0.0001
Hypertension	0.9	1	0.8–0.9	<0.0001	0.8	0.9	0.8–0.9	<0.0001
Liver Disease	1.3	1.1	1.1–1.5	<0.001	--	—	--	--
Renal Disease	1.4	1.1	1.3–1.5	<0.0001	1.4	1.3	1.3–1.5	<0.0001
Ischemic Heart Disease	--	—	--	--	1.1	1.1	1.1–1.2	<0.001
Obesity	0.8	0.9	0.8–0.9	<0.0001	0.9	0.9	0.9–1.0	0.01

* P value <0.05 indicates statistical significance.

Table S5. Multivariable model for 30-day and 1-year all-cause mortality after PSM based on history of SUD at any time.

	**30-day All-cause Mortality**				**1-year All-cause Mortality**			
**Variable, n (%)**	**Odds Ratio**	**Relative Risk**	**OR 95% CI**	**OR P value***	**Odds Ratio**	**Relative Risk**	**OR 95% CI**	**OR P value***
All Time Substance Use	1.7	1.7	1.3–2.1	<0.0001	1.3	1.2	1.2–1.4	<0.0001
Age	1.1	1.1	1.1–1.1	<0.0001	1.1	1.1	1.0–1.0	<0.0001
Female	—	—	—	—	0.9	0.9	0.8–1.0	0.009
Alcohol Use	—	—	—	—	0.7	0.8	0.6–0.9	0.002
Elixhauser Comorbidity Index	—	—	—	—	1.0	1.0	1.0–1.0	0.002
Cancer	2.1	2.1	1.5–3.3	<0.0001	1.7	1.5	1.4–2.0	<0.0001
Cardiac Dysrhythmia	—	—	—	—	1.2	1.1	1.1–1.3	<0.001
COPD	—	—	—	—	1.4	1.3	1.3–1.5	<0.0001
Diabetes Type 1 or 2	0.7	0.7	0.5–0.9	0.003	--	—	--	--
Hypertension	—	—	—	—	0.8	0.9	0.8–0.9	<0.0001
Liver Disease	2.1	2.1	1.3–3.5	0.004	1.6	1.5	1.4–2.0	<0.0001
Renal Disease	1.5	1.5	1.2–2.0	<0.001	1.4	1.3	1.3–1.5	<0.0001
Sleep Apnea	—	—	—	—	0.8	0.8	0.7–0.9	<0.001
Ischemic Heart Disease	—	—	—	—	1.1	1.1	1.0–1.2	0.008
Obesity	0.6	0.6	0.4–0.8	0.003	0.8	0.8	0.7–0.8	<0.0001

* P value <0.05 indicates statistical significance.

Table S6. All-cause and HF-related hospital readmissions, and death of cohort after PSM based on history of SUD at any time and patients with one full year follow-up.

Outcome	Substance Use at Any time, n (%)	No History of Substance Use, n (%)	Odds Ratio	Relative Risk	95% OR CI	OR P-value*
**30-day All-cause Readmission**	1,848 (20.8%)	1,305 (14.7%)	1.5	1.4	1.4–1.6	<0.0001
**30-day HF-related Readmission**	567 (6.4%)	396 (4.4%)	1.5	1.4	1.3–1.7	<0.0001
**180-day All-cause Readmission**	4,722 (53.0%)	3,644 (40.9%)	1.6	1.3	1.5–1.7	<0.0001
**180-day HF-related Readmission**	1,782 (20.0%)	1,386 (15.6%)	1.4	1.3	1.3–1.5	<0.0001
**1-year All-cause Readmission**	5,964 (67.0%)	4,823 (54.2%)	1.7	1.2	1.6–1.8	<0.0001
**1-year HF-related Readmission**	2,476 (27.8%)	1,984 (22.3%)	1.3	1.2	1.3–1.4	<0.0001
**30-day Mortality**	170 (1.9%)	81 (0.9%)	2.1	2.1	1.6–2.8	<0.0001
**1-year Mortality**	782 (8.8%)	509 (5.7%)	1.6	1.5	1.4–1.8	<0.0001

* P value <0.05 indicates statistical significance.

Table S7. All-cause and HF-related hospital readmissions, and death of cohort after PSM based on subgroup with previous history or current SUD and one full year of follow-up.

Outcome	Substance Use Prior/At HF Admission, n (%)	No History of Substance Use, n (%)	Odds Ratio	Relative Risk	95% OR CI	OR P value*
**30-day All-cause Readmission**	968 (17.9%)	833 (15.4%)	1.2	1.2	1.1–1.3	0.0005
**30-day HF-related Readmission**	297 (5.5%)	264 (4.9%)	1.1	1.2	1.0–1.3	0.1527
**180-day All-cause Readmission**	2,552 (47.1%)	2,307 (42.5%)	1.2	1.2	1.1–1.3	<0.0001
**180-day HF-related Readmission**	946 (17.4%)	899 (16.6%)	1.1	1.1	1.0–1.2	0.2297
**1-year All-cause Readmission**	3,384 (62.4%)	3,061 (56.5%)	1.3	1.1	1.2–1.4	<0.0001
**1-year HF-related Readmission**	1,354 (25.0%)	1,300 (24.0%)	1.1	1.1	1.0–1.2	0.2278
**30-day Mortality**	153 (2.8%)	59 (1.1%)	2.6	2.4	1.9–3.6	<0.0001
**1-year Mortality**	526 (9.7%)	334 (6.2%)	1.6	1.6	1.4–1.9	<0.0001

* P value <0.05 indicates statistical significance.

Table S8. All-cause and HF-related hospital readmissions, and death of cohort after PSM based on history of polysubstance or mono-substance use at any time.

	**Substance Use at Any Time (n=11611)**	**Polysubstance Use (n=1829)**	**Monosubstance Use (n=9782)**	**P Value***	**Odds Ratio**	**95% CI**
**30-day Hospital Readmission, n (%)**						
**All-cause**	2454(21.1%)	465(25.4%)	1989(20.3%)	<0.0001	1.3	1.2-1.5
**HF-related**	772(6.6%)	160(8.7%)	612(6.3%)	<0.0001	1.4	1.2-1.7
**180-day Hospital Readmission, n (%)**						
**All-cause**	6350(54.7%)	1127(61.6%)	5223(53.4%)	<0.0001	1.4	1.3-1.6
**HF-related**	2485(21.4%)	467(25.5%)	2018(20.6%)	<0.0001	1.3	1.2-1.5
**1-year Hospital Readmission, n (%)**						
**All-cause**	7881(67.9%)	1325(72.4%)	6556(67%)	<0.0001	1.3	1.2-1.4
**HF-related**	3345(28.8%)	605(33.1%)	2740(28%)	<0.0001	1.3	1.1-1.4
**Mortality, n (%)**						
**30-day Mortality**	191(1.6%)	22(1.2%)	169(1.7%)	0.11	0.7	0.4-1.1
**180-day Mortality**	681(5.9%)	95(5.2%)	586(6.0%)	0.18	0.9	0.7-1.1
**1-year Mortality**	1793(15.4%)	254(13.9%)	1539(15.7%)	0.04	0.9	0.7-1.0

* P value <0.05 indicates statistical significance.

Table S9. Baseline characteristics after PSM based on subgroup of methamphetamine and other stimulant use.

**Baseline Characteristics**	**Methamphetamine and Other Stimulant Use at Any Time (n=968)**	**No Substance Use (n=968)**	**Standardized Differences after Matching**
**Age,** mean ± SD	61.0 (±11.6)	60.9 (±13.4)	0.003
**Female Sex,** n (%)	320 (33.1%)	331 (34.2%)	0.024
**Elixhauser Comorbidity Index,** mean ± SD	0.6 (±2.8)	0.5 (±2.4)	0.062
**Comorbid Conditions,** n (%)			0.05
Diabetes Type 1 or 2	426 (44.0%)	450 (46.5%)	0.009
Hypertension	334 (34.5%)	338 (34.9%)	0.017
Hyperlipidemia	476 (49.2%)	484 (50.0%)	0.023
Ischemic Heart Disease	478 (49.4%)	489 (50.5%)	0.011
Cardiac Dysrhythmia	301 (31.1%)	296 (30.6%)	0.046
Stroke	15 (1.5%)	10 (1.0%)	0.01
COPD	396 (40.9%)	401 (41.4%)	0.043
Asthma	53 (5.5%)	44 (4.5%)	0.01
Renal Disease	461 (47.6%)	456 (47.1%)	0.089
Liver Disease	72 (7.4%)	51 (5.3%)	0.008
Cancer	19 (2.0%)	18 (1.9%)	0.006
Anemia	27 (2.8%)	26 (2.7%)	0.025
Obesity	218 (22.5%)	208 (21.5%)	0.011
Sleep Apnea	173 (17.9%)	169 (17.5%)	0.002
Tobacco Use Disorder	401 (41.4%)	400 (41.3%)	0.079
Alcohol Use	82 (8.5%)	62 (6.4%)	0.01

[a] COPD: chronic obstructive pulmonary disease, SD: standard deviation

Table S10. Readmissions with heart failure or substance use primary diagnosis in the substance use at any time matched cohort.

Readmission Type	Number of Readmissions	No Substance Use (n=11,611)	Substance Use at Any Time (n=11,611)	Total (N=23,222)
**30-day HF-related Readmissions**	0	11,090 (95.5%)	10,839 (93.4%)	21,929 (94.4%)
	1	497 (4.3%)	711 (6.1%)	1,208 (5.2%)
	≥2	24 (0.2%)	61 (0.5%)	85 (0.4%)
**1-year HF-related Readmissions**	0	8,950 (77.1%)	8,266 (71.2%)	17,216 (74.1%)
	1	1,822 (15.7%)	2,119 (18.2%)	3,941 (17.0%)
	≥2	839 (7.2%)	1,226 (10.6%)	2,065 (8.9%)
**30-day Substance-related Readmissions**	0	11,611 (100.0%)	11,601 (99.9%)	23,212 (100.0%)
	1	0 (0.0%)	10 (0.1%)	10 (<0.1%)
**1-year Substance-related Readmissions**	0	11,611 (100.0%)	11,540 (99.5%)	23,151 (99.7%)
	1	0 (0.0%)	56 (0.5%)	56 (0.2%)
	≥2	0 (0.0%)	5 (<0.1%)	5 (<0.1%)

Table S11. Readmissions with heart failure or substance use primary diagnosis in the prior/current substance use matched cohort.

Readmission Type	Number of Readmissions	No Substance Use (n=7,069)	Substance Use Prior/At HF Admission (n=7,069)	Total (N=14,138)
**30-day HF-related Readmissions**	0	6,739 (95.3%)	6,669 (94.3%)	13,408 (94.8%)
	1	311 (4.4%)	379 (5.4%)	690 (4.9%)
	≥2	19 (0.3%)	21 (0.3%)	40 (0.3%)
**1-year HF-related Readmissions**	0	5,352 (75.7%)	5,239 (74.1%)	10,591 (74.9%)
	1	1,178 (16.7%)	1,213 (17.2%)	2,391 (16.9%)
	≥2	539 (7.6%)	617 (8.7%)	1,156 (8.2%)
**30-day Substance-related Readmissions**	0	7,068 (100.0%)	7,068 (100.0%)	14,136 (100.0%)
	1	1 (<0.1%)	1 (<0.1%)	2 (<0.1%)
**1-year Substance-related Readmissions**	0	7,065 (99.9%)	7,055 (99.8%)	14,120 (99.9%)
	1	4 (0.1%)	13 (0.2%)	17 (0.1%)
	≥2	0 (0.0%)	1 (<0.1%)	1 (<0.1%)

Table S12. Readmissions with heart failure or substance use primary diagnosis in the methamphetamine and other stimulant use at any time matched cohort.

Readmission Type	Number of Readmissions	No Substance Use (n=968)	Methamphetamine and Other Stimulant Use at Any Time (n=968)	Total (N=1,936)
**30-day HF-related Readmissions**	0	921 (95.1%)	890 (91.9%)	1,811 (93.5%)
	1	45 (4.6%)	67 (6.9%)	112 (5.8%)
	≥2	2 (0.2%)	11 (1.1%)	13 (0.7%)
**1-year HF-related Readmissions**	0	723 (74.7%)	645 (66.6%)	1,368 (70.7%)
	1	162 (16.7%)	182 (18.8%)	344 (17.8%)
	≥2	83 (8.6%)	141 (14.6%)	224 (11.6%)
**30-day Substance-related Readmissions**	0	968 (100.0%)	967 (99.9%)	1,935 (99.9%)
	1	0 (0.0%)	1 (0.1%)	1 (0.1%)
**1-year Substance-related Readmissions**	0	968 (100.0%)	952 (98.3%)	1,920 (99.2%)
	1	0 (0.0%)	13 (1.3%)	13 (0.7%)
	≥2	0 (0.0%)	3 (0.3%)	3 (0.2%)

Figure S1. Love Plot of Covariate Balance Before and After Propensity Score Matching for History of Substance Use at Any Time

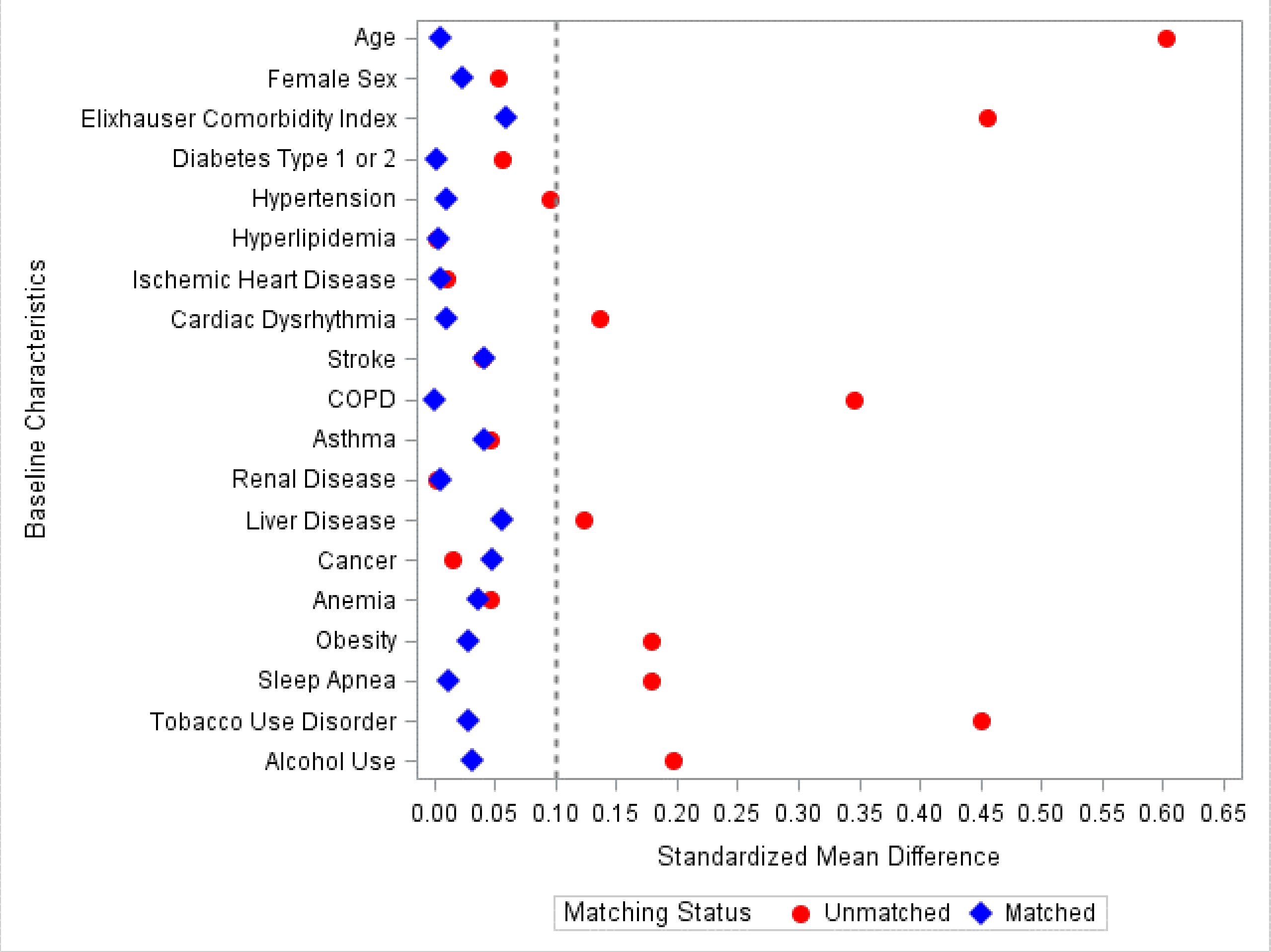

Figure S2. Love Plot of Covariate Balance Before and After Propensity Score Matching for Prior or Current Substance Use

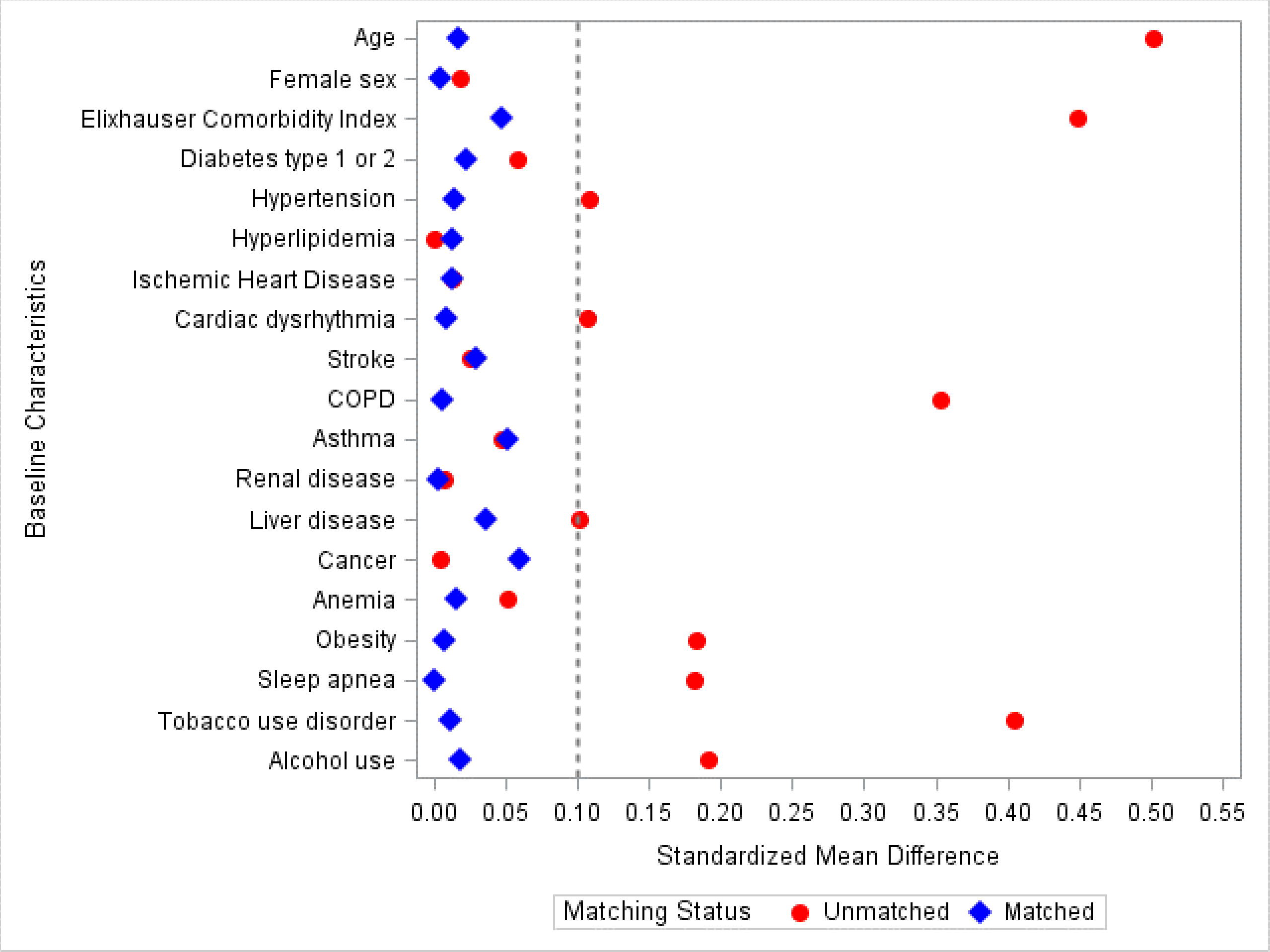

Figure S3. Love Plot of Covariate Balance Before and After Propensity Score Matching for History of Methamphetamine Use at Any Time

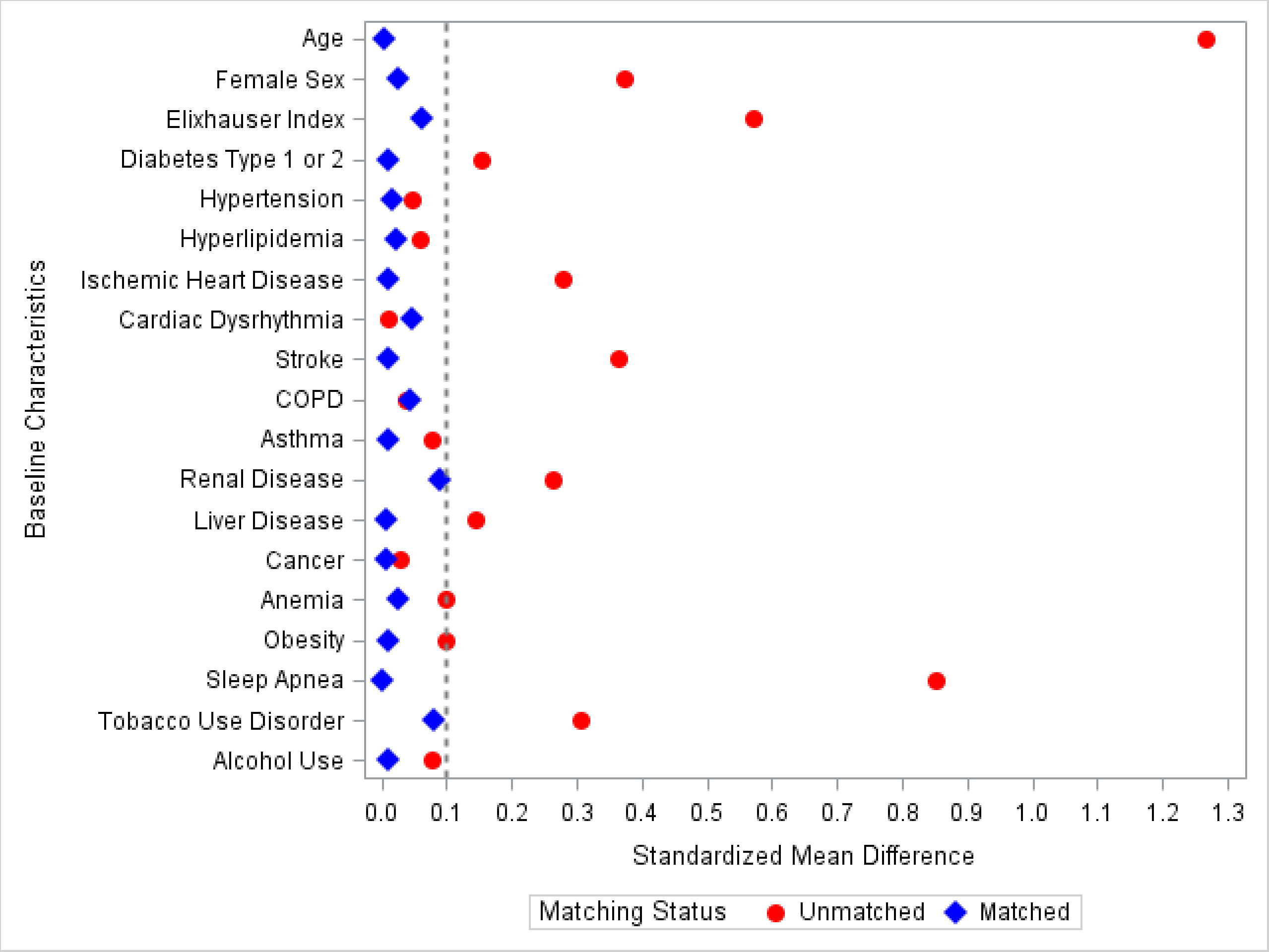

Supplement: Supplementary file 1 [file jcm-15-06974-s001.zip › jcm-4514108-supplementary.pdf]
